# Utility of the new cobas HCV test for viral load monitoring during direct-acting antiviral therapy

**DOI:** 10.1371/journal.pone.0224751

**Published:** 2019-11-18

**Authors:** Marcus M. Mücke, Benjamin Maasoumy, Julia Dietz, Victoria T. Mücke, Christian O. Simon, Jesse A. Canchola, Marcus Cornberg, Ed G. Marins, Michael P. Manns, Stefan Zeuzem, Heiner Wedemeyer, Christoph Sarrazin, Johannes Vermehren

**Affiliations:** 1 Department of Internal Medicine 1, University Hospital Frankfurt, Frankfurt am Main, Germany; 2 Department of Gastroenterology, Hepatology and Endocrinology, Hannover Medical School, Hannover, Germany; 3 Roche Molecular Systems, Pleasanton, CA, United States of America; 4 Department of Gastroenterology and Hepatology, University Hospital Essen, Essen, Germany; 5 Department of Gastroenterology, St. Josefs-Hospital, Wiesbaden, Germany; National Taiwan University Hospital, TAIWAN

## Abstract

**Background:**

The COBAS AmpliPrep/COBAS TaqMan assay HCV (CAP/CTM) is widely used in clinical routine for HCV testing. Recently, the new cobas HCV test was established for high throughput testing with minimal operator intervention. As different assays may yield different quantitative/qualitative results that possibly impact treatment decisions, the aim of this study was to externally evaluate the cobas HCV test performance in comparison to CAP/CTM in a clinically relevant setting.

**Methods:**

Serum samples were obtained from 270 patients who received direct acting antiviral therapy with different treatment regimens at two study sites (Hannover and Frankfurt) in 2016. Overall, 1545 samples (baseline, on-treatment and follow-up) were tested in parallel by both assays.

**Results:**

The mean difference between cobas HCV and CAP/CTM for the quantification of HCV RNA was 0.008 log_10_ IU/ml HCV RNA (95% limits of agreement: -0.02–0.036) showing excellent agreement of both assays. With respect to clinical cut offs (HCV RNA detectable vs. target not detected and HCV RNA above the lower limit of quantification (LLOQ) vs. <LLOQ), discordant results were obtained in 9.5% and 4.6%, respectively; the greatest differences were observed during early stages of antiviral therapy (week 1, week 2 and week 4), but none were statistically significant. Overall percent agreement for SVR between cobas HCV and CAP/CTM at the 15 IU/ml cutoff was 99.2% (95%CI 92.7%-100%).

**Conclusion:**

The performance of the new cobas HCV test was comparable to CAP/CTM in a clinical setting representing a large patient population with HCV GT 1 and 3 treated with DAAs.

## Introduction

Chronic hepatitis C virus (HCV) infection is a major global health problem with approximately 70 million infected individuals worldwide who are at risk of significant morbidity and mortality [1, 2]. Following the introduction of direct-acting antivirals (DAAs), viral eradication can now be achieved in almost every patient, even in those earlier considered difficult-to-treat, including those with decompensated cirrhosis, renal impairment or active drug abuse [3–5]. The ultimate goal of antiviral therapy is to achieve a sustained virological response (SVR), which is considered a cure and associated with reduced liver inflammation, fibrosis regression, increased quality of life and overall survival [6–8].

SVR is defined as persistent undetectable HCV RNA 12 weeks after the end of therapy (FU12), assessed with a highly sensitive assay with a lower limit of detection (LOD) of ≤ 15 UI/ml [9]. On-treatment HCV RNA has been commonly measured as a secondary endpoint in initial clinical trials investigating efficacy of DAA regimens[10], as it was used for “response-guided therapy” in patients treated with pegylated interferon and first-generation protease inhibitors. Although measurements of HCV RNA are still recommended at specific time-points during antiviral therapy by current clinical practice guidelines[9], they are no longer used to tailor treatment duration with DAAs, and in many cases have been even abandoned in daily clinical practice. This highlights the importance of highly sensitive and reliable HCV RNA assays, as the only measurement after initiation of DAA therapy may be confirmation of SVR 12 weeks after the end of therapy.

In most clinical trials, the Roche COBAS® TaqMan HCV test version 2 for use with the manual High Pure System extraction kit (HPS/CTM) was used to determine HCV RNA[11–13] with a lower limit of quantification of 25 IU/ml and a LOD of 20 IU/ml, according to the United States Food and Drug Administration (FDA) label [14]. Since then, several fully automated molecular assays have been implemented in clinical routine, including the COBAS® AmpliPrep/COBAS® TaqMan HCV test version 2.0 (CAP/CTM) and the Abbott RealTime HCV assays, both with LLOQ and LODs of 15IU/ml and 12IU/ml, respectively [15, 16].

In a previous study we showed that CAP/CTM and HPS/CTM showed significantly different response rates during the early stages of DAA therapy. Yet on-treatment response was not predictive of SVR with either assay and these differences disappeared at later stages during treatment and test results were in excellent agreement[17].

More recently, the new cobas® HCV test for use on the cobas 6800/8800 systems (cobas HCV) with a LOD and LLOQ of 15 IU/ml has been introduced and is rapidly replacing CAP/CTM in laboratory routine[18]. Yet, to date, no external clinical performance evaluation has been published. The aim of this study was to validate and correlate the performance of cobas HCV with the established CAP/CTM in a large patient population with HCV genotypes (GT) 1 and 3 treated with DAAs.

## Materials and methods

Blood samples were obtained from two study sites at the University Hospital Frankfurt, Germany, and the Hannover Medical School, Hanover, Germany in 2016.

For inclusion in this study, specimens must have been collected from patients who were older than 18 years of age at the time of collection. Patients were infected with HCV GTs 1 or 3 and were treated with one of the following antiviral regimens: a fixed dose combination of 90mg ledipasvir and 400mg sofosbuvir (LDV/SOF) ± weight-based ribavirin (RBV), a fixed dose combination of 25/150/100mg ombitasvir/paritaprevir/ritonavir and 500mg dasabuvir (PrOD) ± weight-based RBV, a combination of 60mg daclatasvir and 400mg sofosbuvir (DCV/SOF) ± weight-based RBV, or 400mg sofosbuvir plus weight-based RBV (SOF/RBV). Patient demographics, including age, gender, HCV GT, treatment regimen, and presence of cirrhosis were anonymously and retrospectively collected from electronic hospital charts. The ethics committee of the University Hospital Frankfurt and Hannover Medical School approved the initial study. All patients provided informed written consent for sample usage and data retrieval from their medical records. For the usage of left over samples no additional ethic statement was necessary and all samples were fully anonymized.

### HCV RNA measurements

Specimens were tested with the new cobas® HCV test (cobas HCV) at Hannover Medical School and with the COBAS® AmpliPrep/ COBAS® TaqMan® v2 HCV test (CAP/CTM) at University Hospital Frankfurt and Hannover Medical School, and results were compared between assays.

CAP/CTM has a lower limit of detection (LOD) of 15IU/ml and a lower limit of quantification (LLOQ) of 15 IU/ml according to the label [16]. The new cobas HCV test is a dual-probe test with a LOD of 15 IU/ml and a LLOQ of 15 IU/ml[18].

For each patient, at least 4 time points were selected for this study—including one before treatment, two to six time points during therapy, and one after treatment (i.e., 12 weeks after end of treatment). All patient specimens to be used in this study were selected from archived samples that had been stored at -20°C or colder for no more than 3 years. On-treatment HCV RNA levels were compared using two categorical cutoffs: a) ≥LLOQ vs. <LLOQ/detectable and b) detectable vs. target not detected (TND), both of which are widely used clinically.

### Statistical analysis

All statistical analyses were carried out using the SAS System v9.3 or higher, via the SAS Enterprise Guide v5.1 or higher (SAS Institute, Cary, NC) graphical user interface. All confidence intervals (CIs) were constructed using a 95% confidence level. Assay comparisons were made using Deming regression and Bland Altman plot analyses. Outlier testing was conducted using the method of externally Studentized residuals. A statistical outlier was identified if the absolute value of the externally Studentized residual was greater than 4. Outcome data in subgroups were analyzed by Fisher’s exact test. Differences in on-treatment response between assays were compared using the McNemar’s test. Differences were considered significant at p ≤ 0.05.

## Results

### Cohort characteristics

Overall, 1979 samples from 270 patients with chronic hepatitis C were tested. More than half of the patients were treatment-naïve (n = 156, 57.8%) and 120 patients (44.4%) had compensated liver cirrhosis. Two hundred and two patients were infected with HCV GT1 (74.8%) and 68 patients had HCV GT3 (25.2%). DAA treatments were conducted according to the guidelines at the time of the study: a total of 154 patients received LDV/SOF±RBV, 37 patients received PrOD±RBV, 47 patients DCV/SOF±RBV and 32 patients were treated with SOF/RBV. Median treatment duration was 12 weeks (n = 46 were treated for 8 weeks, n = 140 were treated for 12 weeks, n = 1 for 16 weeks, and n = 83 patients received 24 weeks of therapy). The overall SVR rate was 96.7%. HCV RNA levels could be tested in 1545 samples with both assays and valid test results were used for the current analysis.

### Comparison of HCV RNA levels measured with cobas HCV and CAP/CTM in patients with quantifiable viral loads

The overlapping range of both assays with quantifiable HCV RNA included 447 samples. Five paired samples for which one or both test results fell outside the linear range were not included in the analysis, but are displayed in Table 1.

**Table 1:** Sample listing for cobas HCV in samples with CAP/CTM target not detected (TND); n = 5.

Subject ID	Time Point		cobas HCV Result (IU/mL)	cobas HCV Result (log10 IU/mL)	CAP/CTM (IU/mL) ^a^	CAP/CTM Result Category (IU/mL)
10740.14	Week 4		16.41	1.215	0	TND
1968	FU12		687.54	2.837	0	TND
3011	Week 4		16.11	1.207	0	TND
4032.15	Week 2		19.65	1.293	0	TND
7285.14	Week 24		31.35	1.496	0	TND

Abbreviations: COBAS® AmpliPrep/COBAS® TaqMan HCV test version 2.0, CAP/CTM; identification, ID; hepatitis C virus, HCV; target not detected, TND; follow-up after 12 weeks, FU12

The patients with the ID 10740.14, 3011, 4032.15 and 7285.14 were TND in the next measurement in both tests and all achieved SVR. The patient with the ID 1968 viral load was TND in both tests in two prior measurements. Unfortunately, no further testing at FU 12 was possible due to lack of material.

Additionally, the statistical outlier analysis yielded one outlier (Sample ID 2048; CAP/CTM result: 6.5198 log_10_ IU/ml and cobas HCV result: 2.2244 log_10_ IU/ml; Studentized residual -22.3073). The sample was from a GT 1 patient who was successfully treated with LDV/SOF for 8 weeks. The baseline sample was the statistical outlier; test results of samples thereafter (week 4, week 8 and FU 12) were in agreement.

**Fig 1:**
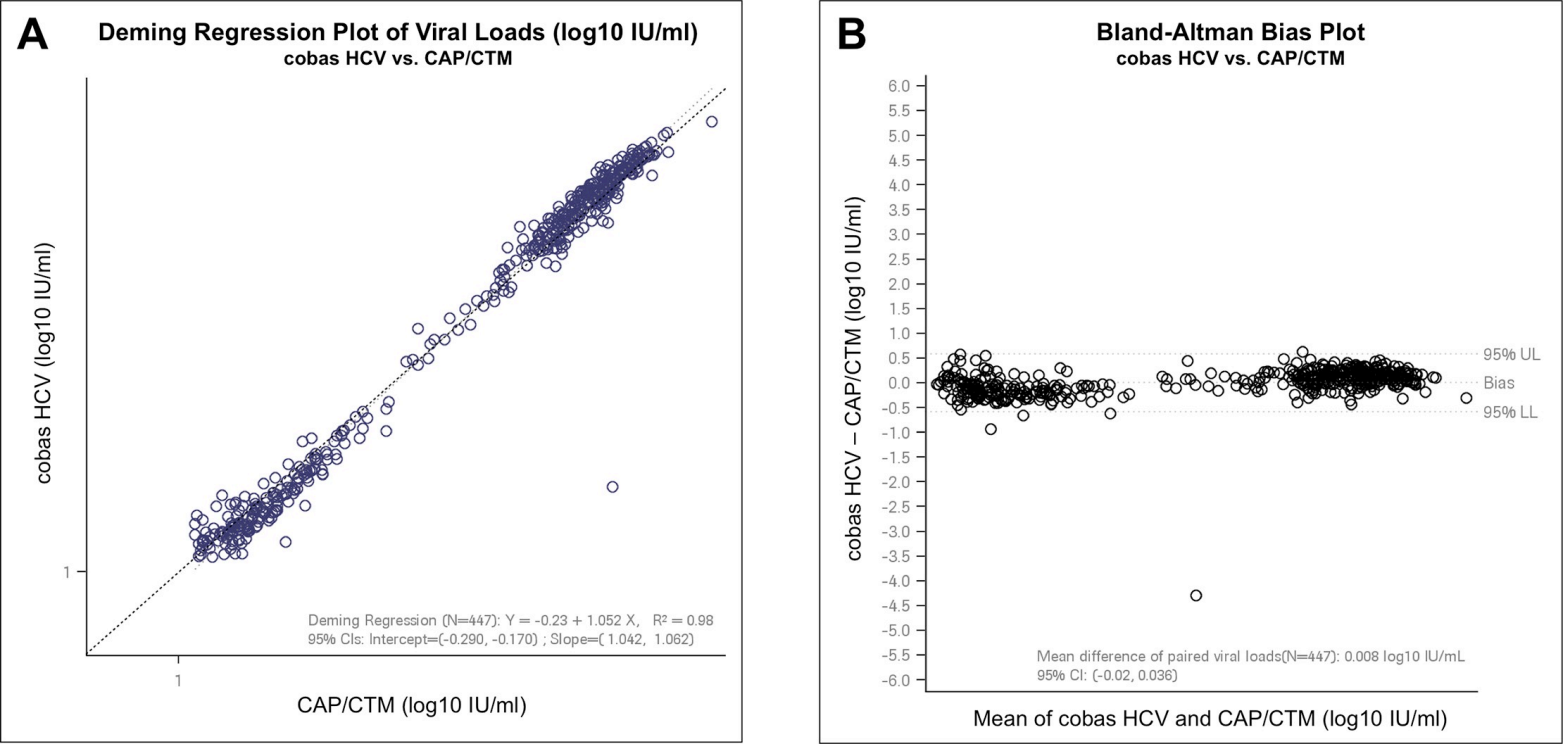
Deming Regression Plot of Viral Loads (log_10_ IU/mL, A) and Bland-Altman bias plot (B) for cobas HCV vs. CAP/CTM (including one statistical outlier).

Fig 1A shows the scatterplot of the Y result versus the X result presented with the Deming Regression line of the Y_i_ versus the X_i_ results for each valid assay pair (n = 447), including the identified outlier. Based on Deming regression analysis, the coefficient of determination among valid samples was R^2^ = 0.98. Bland Altman plot analysis revealed that the mean difference between cobas HCV and CAP/CTM was 0.008 log_10_ IU/ml HCV RNA (95% limits of agreement: -0.02 and 0.036; e.g. 1.02 IU/ml, 95% CI 0.96–1.09) reflecting excellent agreement of both assays.

### Comparison of cobas HCV and CAP/CTM using clinical cutoffs: ≥LLOQ vs. <LLOQ and detectable HCV RNA vs. TND at different time points during DAA therapy

Serum samples were available for testing with both assays from 64, 184, 196, 168, 198, 70, and 252 patients at treatment week 1, week 2, week 4, week 8, week 12, week 24 and 12 weeks after end of treatment (FU12), respectively. The remaining samples were from baseline or other, non-standardized time points during antiviral therapy. Among 1132 samples tested at standardized time points during DAA treatment, 108 (9.5%) and 52 (4.6%) had discordant results with respect to detectable vs. TND and ≥LLOQ vs. <LLOQ, respectively. Test results for each treatment week and FU12 with concordant and discordant results between both cobas HCV and CAP/CTM are presented in S1–S7 Tables in detail.

**Fig 2:**
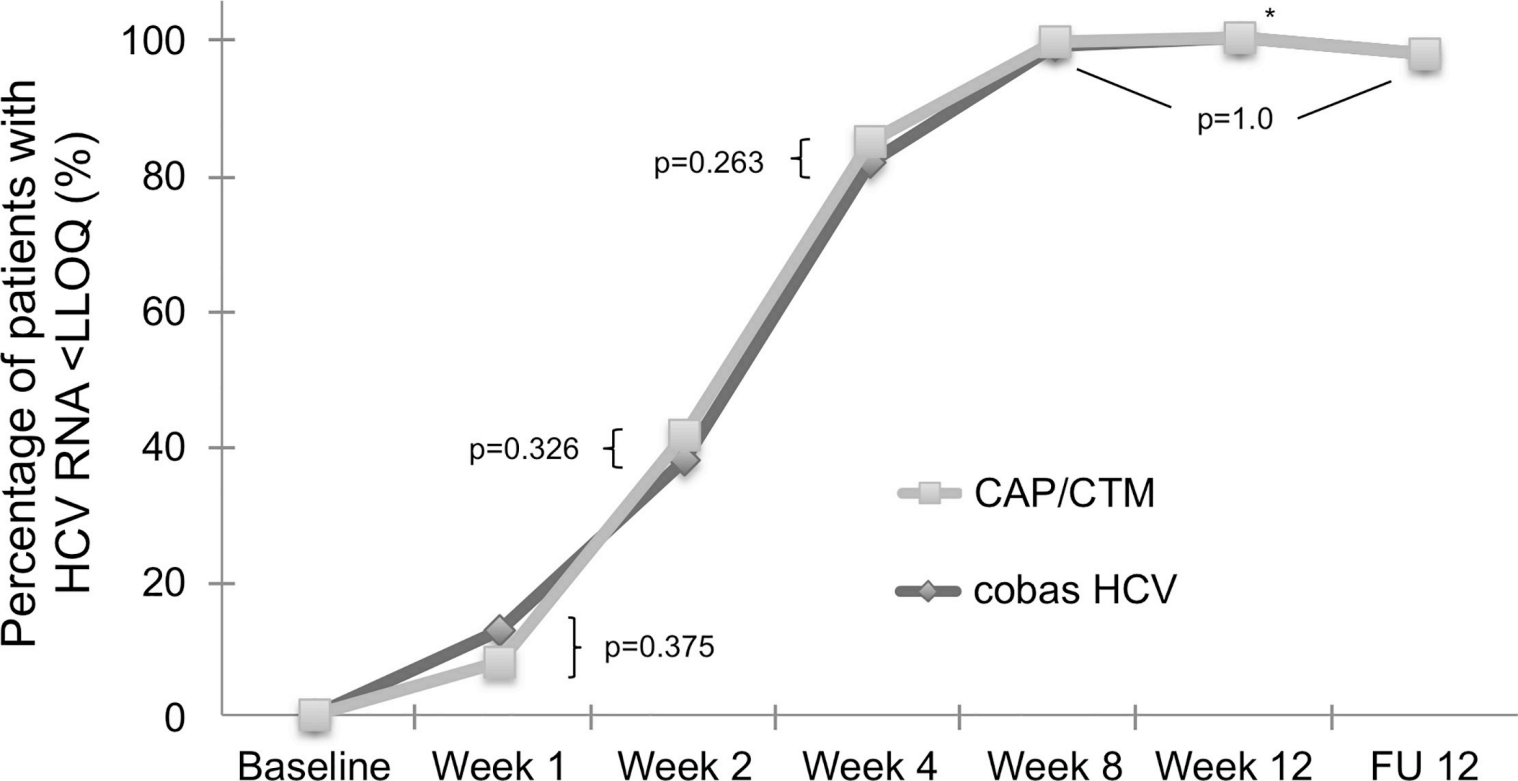
Virologic responses at week 1, week 2, week 4, week 8, week 12 and 12 weeks after the end of treatment (FU12) according to cobas HCV and the CAP/CTM. There was no significant difference between the two assays. *As there are no discordant pairs, p-value cannot be calculated.

The proportion of samples at week 1 that was <LLOQ was higher when measured with the new cobas HCV as compared to CAP/CTM (12.5% vs. 7.8%). However this difference was not statistically significant. At all other on-treatment time points the proportion of samples that was <LLOQ was lower or comparable to CAP/CTM when measured with cobas HCV, and there were no significant differences between test results (Table 2, Fig 2).

**Table 2:** Comparison of virologic responses at week 1,2,4,8,12, and at 12 weeks after the end of treatment (FU12) according to cobas HCV and CAP/CTM, respectively.

	cobas HCV		CAP/CTM		p-value
	< LLOQ	≥LLOQ	< LLOQ	≥LLOQ	
**Week 1** (n = 64), n(%)	8 (12.5)	56 (87.5)	5 (7.8)	59 (92.2)	0.375
**Week 2** (n = 186), n(%)	70 (37.6)	116 (62.4)	77 (41.4)	109 (58.6)	0.324
**Week 4** (n = 196), n(%)	160 (81.6)	36 (18.4)	166 (84.7)	30 (15.3)	0.263
**Week 8** (n = 168), n(%)	166 (98.8)	2 (1.2)	167 (99.4)	1 (0.6)	1.0
**Week 12** (n = 198), n(%)	198 (100)	0 (0)	198 (100)	0 (0)	NA*
**FU 12** (n = 252), n(%)	246 (97.6)	6 (2.4)	246 (97.6)	6 (2.4)	1.0

*As there are no discordant pairs, p-value cannot be calculated. Virologic responses were defined as below the lower limit of quantification (<LLOQ: <15 IU/ml for both assays) and target not detected (TND).

During early treatment (week 1, week 2 and week 4) there were 4, 15, and 7 patient samples with HCV RNA <LLOQ according to cobas HCV but quantifiable HCV RNA according to CAP/CTM. CAP/CTM HCV RNA results ranged from 16 to 29 IU/ml, 19 to 98 IU/ml and 17 to 26 IU/ml, respectively. At later time points, there was only one sample with HCV RNA <LLOQ with cobas HCV but quantifiable HCV RNA according to CAP/CTM. Overall percent agreement (OPA) of SVR for cobas and CAP/CTM at the 15 IU/ml cutoff for patients with a sample taken at follow-up week 12 after therapy was 99.2% (95%CI 92.7%-100%).

### Comparison of cobas HCV and CAP/CTM for the prediction of SVR

**Fig 3:**
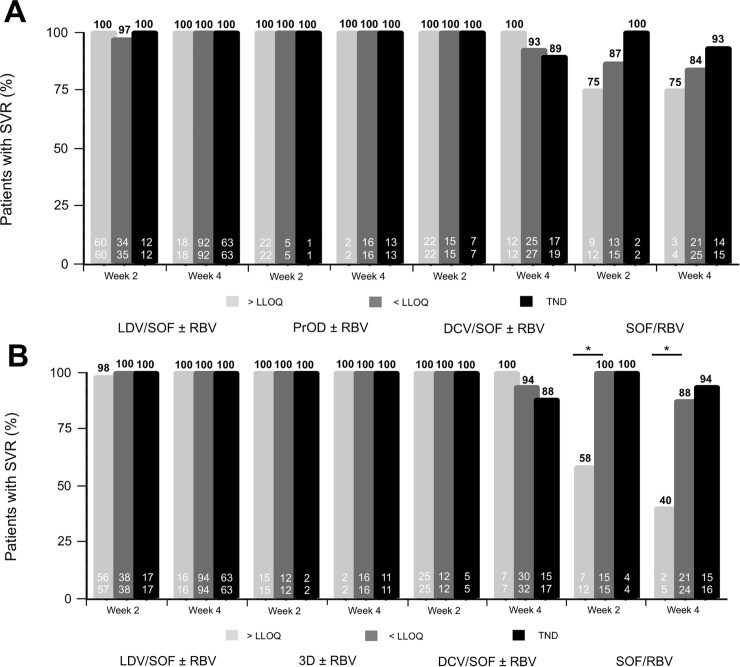
**Sustained virologic response (SVR) rates according to early HCV RNA response at treatment week 2 and week 4 with different DAA regimens assessed with (A) cobas HCV and (B) CAP/CTM.** SVR rates were not significantly different between patients with quantifiable (≥LLOQ) vs. non-quantifiable (<LLOQ and TND) and detectable (≥LLOQ and <LLOQ) vs. undetectable (TND) HCV RNA in the three most commonly used DAA regimens (LDV/SOF, PrOD, DCV/SOF) according to both assays. SVR rates in patients with quantifiable (≥LLOQ) vs. non-quantifiable (<LLOQ and TND) in the SOF/RBV treatment group showed significant difference at week 2 (p = 0.01) and week 4 (p<0.05) when applying CAP/CTM but not with cobas HCV.

Sustained virologic response (SVR) rates according to early HCV RNA response at week 2 and week 4 with different treatment regimens assessed with cobas HCV and CAP/CTM are depicted in Fig 3. Of note, SVR rates were not significantly different between patients with quantifiable (≥LLOQ) vs. non-quantifiable (<LLOQ and TND) and detectable ((≥ LLOQ and <LLOQ) vs. undetectable (TND) HCV RNA in different DAA combination treatments (LDV/SOF, PrOD, DCV/SOF) according to both assays. Interestingly, SVR rates in patients with quantifiable (≥LLOQ) vs. non-quantifiable (<LLOQ or TND) in the SOF/RBV treatment group were significantly different at week 2 (p = 0.01) and week 4 (p<0.05) when applying CAP/CTM, that is, patients with quantifiable HCV RNA at week 2/4 had significantly lower SVR rates. This was not the case with the cobas HCV assay where on-treatment HCV RNA levels had no predictive value for SVR.

## Discussion

In our study, the new fully automated cobas HCV assay for use on the 6800 platform was in excellent agreement with the already established CAP/CTM HCV test for the quantification of HCV RNA at baseline, during and after DAA therapy in all quantifiable samples. There were only five discordant samples, all of which had quantifiable HCV RNA according to cobas HCV, while CAP/CTM did not detect HCV RNA.

This is the first large scale, multi-center study to report an external validation of the new cobas HCV assay that will replace most CAP/CTM platforms in the near future and applying clinical relevant endpoints.

In our study, using clinical cut offs, only 9.5% and 4.6% of all samples tested had discordant results with respect to detectable vs. TND and ≥LLOQ vs. <LLOQ, respectively. Of note, discordant results were mostly observed during early antiviral therapy. However, these differences were not statistically significant and disappeared over the course of therapy, as more samples became undetectable. Similar observations were made earlier when CAP/CTM was compared with the HPS/CTM assay, as well as in a comparison study of CAP/CTM with the Abbott RealTime HCV assay, which might reflect a typical phenomenon when more sensitive assays were introduced [17, 19].

Overall percentage agreement of SVR for cobas HCV and CAP/CTM at the 15 IU/ml cutoff was 99.2%. Interestingly, each assay found detectable HCV RNA in one patient sample at FU12, while the other did not. However, the clinical impact of residual viremia during follow-up may be negligible [20].

In the present study, we also investigated whether early treatment response measured with cobas HCV may be useful for predicting SVR. As shown earlier[17], the CAP/CTM had no predictive implication in patients treated with LDV/SOF, PrOD or DCV/SOF, and comparable results were now observed for cobas HCV. In patients treated with SOF/RBV, we observed a numerically lower SVR rate in patients with quantifiable HCV RNA at both treatment weeks 2 and 4. However, as this treatment regimen has been replaced by newer more potent DAA regimens the clinical relevance may be low. Overall, the use of on-treatment HCV measurements for response prediction has diminished with the introduction of highly potent pan-genotypic DAA regimens, as SVR can now be achieved in almost every patient [10].

For the present study, only patients with GT 1 and 3 were included, as these two GTs are the most prevalent HCV genotypes in Germany. Given the lack of available patient samples from other genotypes. we were unable to perform assay correlations in other GTs (e.g. GT 2). In a US-based study, a good overall correlation between the two tests in samples from patients with various GT was noted. However, the number of included patient samples was low and no GT specific subgroup analyses were performed [21].

Taken together, our study successfully validated the performance of the new cobas HCV test for use in patients treated with DAAs. We showed that cobas HCV was highly comparable with the established CAP/CTM assay in a large patient population infected with the most common HCV genotypes 1 and 3 in Europe.

## Supporting information

S1 Table Comparison of cobas HCV and CAP/CTM using clinical cutoffs: ≥LLOQ vs. <LLOQ and detectable HCV RNA vs. TND at week 1. (DOCX)

S2 Table Comparison of cobas HCV and CAP/CTM using clinical cutoffs: ≥LLOQ vs. <LLOQ and detectable HCV RNA vs. TND week 2. (DOCX)

S3 Table Comparison of cobas HCV and CAP/CTM using clinical cutoffs: ≥LLOQ vs. <LLOQ and detectable HCV RNA vs. TND week 4. (DOCX)

S4 Table Comparison of cobas HCV and CAP/CTM using clinical cutoffs: ≥LLOQ vs. <LLOQ and detectable HCV RNA vs. TND week 8. (DOCX)

S5 Table Comparison of cobas HCV and CAP/CTM using clinical cutoffs: ≥LLOQ vs. <LLOQ and detectable HCV RNA vs. TND week 12. (DOCX)

S6 Table Comparison of cobas HCV and CAP/CTM using clinical cutoffs: ≥LLOQ vs. <LLOQ and detectable HCV RNA vs. TND week 24. (DOCX)

S7 Table Comparison of cobas HCV and CAP/CTM using clinical cutoffs: ≥LLOQ vs. <LLOQ and detectable HCV RNA vs. TND 12 weeks after end of treatment. (DOCX)

S1 File Raw data. (XLSX)

## Abbreviations

HCV: hepatitis C virus
DAA: direct-acting antiviral
SVR: sustained virological response
LOD: lower limit of detection
HPS/CTM: COBAS TaqMan HCV test version 2 for use with the manual High Pure System extraction kit
FDA: Food and Drug Administration
CAP/CTM: COBAS AmpliPrep/COBAS TaqMan assay HCV test version 2.0
LLOQ: lower limit of quantification
GT: genotype
LDV: ledipasvir
SOF: sofosbuvir
RBV: ribavirin
PrOD: ombitasvir/paritaprevir/ritonavir and dasabuvir
DCV: daclatasvir
TND: target not detected
CI: confidence interval
OPA: overall percent agreement
