# Supplementary material for: Utility of the new cobas HCV test for viral load monitoring during direct-acting antiviral therapy
Source: PLoS One. 2019 Nov 18;14(11):e0224751. doi: 10.1371/journal.pone.0224751 (PMC6860929; doi:10.1371/journal.pone.0224751)
Supplement: S3 Table — (DOCX) [file pone.0224751.s003.docx]

**Supplementary Information**

**S3 Table. Comparison of cobas HCV and CAP/CTM using clinical cutoffs: ≥LLOQ vs. <LLOQ and detectable HCV RNA vs. TND week 4.**

| **cobas HCV** | **CAP/CTM** | | | |
| --- | --- | --- | --- | --- |
| **Frequency** | **≥ 15 IU/mL** | **< LLOQ** | **TND** | **Total** |
| **≥ 15 IU/mL** | 23 | 11 | 2 | 36 |
| **< LLOQ** | 4 | 27 | 19 | 50 |
| **TND** | 3 | 21 | 86 | 110 |
| **Total** | 30 | 59 | 107 | 196 |
